# Interrater agreement and variability in visual reading of [18F] flutemetamol PET images

**DOI:** 10.1007/s12149-024-01977-7

**Published:** 2024-09-24

**Authors:** Akinori Takenaka, Takashi Nihashi, Keita Sakurai, Keiji Notomi, Hokuto Ono, Yoshitaka Inui, Shinji Ito, Yutaka Arahata, Akinori Takeda, Kazunari Ishii, Kenji Ishii, Kengo Ito, Hiroshi Toyama, Akinori Nakamura, Takashi Kato, BATON Study Group

**Affiliations:** 1 Department of Radiology, Fujita Health University School of Medicine, Toyoake, Japan; 2 Department of Radiology, National Center for Geriatrics and Gerontology, Obu, Japan; 3 Micron Inc. Imaging Service Dept., Tokyo, Japan; 4 Department of Radiology, Anjo Kosei Hospital, Anjo, Japan; 5 Department of Neurology, National Center for Geriatrics and Gerontology, Obu, Japan; 6 Department of Radiology, Faculty of Medicine, Kindai University, Osakasayama, Japan; 7 Team for Neuroimaging Research, Tokyo Metropolitan Institute of Gerontology, Tokyo, Japan; 8 Department of Clinical and Experimental Neuroimaging, National Center for Geriatrics and Gerontology, Obu, Japan; 9 Department of Biomarker Research, National Center for Geriatrics and Gerontology, Obu, Japan

**Keywords:** Amyloid, Positron emission tomography, Visual rating, Centiloid scale

## Abstract

**Objective:**

The purpose of this study was to validate the concordance of visual ratings of [18F] flutemetamol amyloid positron emission tomography (PET) images and to investigate the correlation between the agreement of each rater and the Centiloid (CL) scale.

**Methods:**

A total of 192 participants, clinically classified as cognitively normal (CN) (*n* = 59), mild cognitive impairment (MCI) (*n* = 65), Alzheimer’s disease (AD) (*n* = 55), or non-AD dementia (*n* = 13), participated in this study. Three experts conducted visual ratings of the amyloid PET images for all 192 patients, assigning a confidence level to each rating on a three-point scale (certain, probable, or neither). The positive or negative determination of amyloid PET results was made by majority vote. The CL value was calculated using the CapAIBL pipeline.

**Results:**

Overall, 101 images were determined to be positive, and 91 images were negative. Of the 101 positive images, the three raters were in complete agreement for 92 images and in disagreement for 9 images. Of the 91 negative images, the three raters were in complete agreement for 75 images and in disagreement for 16 images. Interrater reliability among the three experts was particularly high, with both Fleiss’ kappa and Conger’s kappa measuring 0.83 (0.76–0.89). The CL values of the unanimous positive group were significantly greater than those of the other groups, whereas the CL values of the unanimous negative group were significantly lower than those of the other groups. Images with rater disagreement had intermediate CLs. In cases with a high confidence level, the positive or negative visual ratings were in almost complete agreement. However, as confidence levels decreased, experts’ visual ratings became more variable. The lower the confidence level was, the greater the number of cases with disagreement in the visual ratings.

**Conclusion:**

Three experts independently rated 192 amyloid PET images, achieving a high level of interrater agreement. However, in patients with intermediate amyloid accumulation, visual ratings varied. Therefore, determining positive and negative decisions in these patients should be performed with caution.

## Introduction

The number of dementia patients is increasing as the population ages and is predicted to increase from 57.4 million patients in 2019 to 152.8 million patients in 2050 worldwide [1]. Alzheimer’s disease (AD), a progressive neurodegenerative disorder characterized by the presence of extracellular plaques containing amyloid-β (Aβ) and intracellular neurofibrillary tangles containing tau, is the most common type of dementia.

In January 2023, the U.S. Food and Drug Administration (FDA) approved the use of lecanemab for the treatment of patients with early AD after a large phase III trial revealed statistically significant improvements in cognitive function and clinical endpoints [2]. Lecanemab is a humanized IgG1 monoclonal antibody that treats this progressive, chronic disease by targeting soluble aggregates of Aβ, removing the most neurotoxic Aβ protofibrils that continually accumulate, and eliminating existing plaques. Evidence of Aβ accumulation in the patient’s brain is required to be eligible for lecanemab [3]. Currently, Aβ accumulation can be confirmed by cerebrospinal fluid testing or amyloid positron emission tomography (PET), which can be used for the in vivo detection of brain amyloid plaques.

Three ^18^F-labeled PET tracers (18F-florbetapir, 18F-flutemetamol, and 18F-florbetaben) have been approved by the FDA and the European Medicines Agency. The FDA-approved method for the classification of images as amyloid-positive or amyloid-negative is visual reading [4] [5] [6] [7].

In clinical practice, the interpretation of amyloid PET scans relies on a binary classification (positive/negative) determined through visual assessment. However, inconsistencies in visual assessments caused by varied circumstances (i.e., reader experience) can lead to classification discrepancies, particularly in cases of equivocal findings. Avoiding ambiguous assessments is crucial, especially when determining eligibility for AD-modifying drugs.

The Centiloid (CL) scale was developed to standardize quantitative amyloid imaging measures by scaling the outcome of each analysis method or tracer on a scale from 0 (young controls) to 100 (typical AD patients) [8]. The CL scale enables us to estimate the degree of amyloid accumulation during AD progression across amyloid PET pharmaceuticals.

The objective of this study was to validate the concordance of visual assessments of amyloid PET tracer (18F-flutemetamol) images among three experts involved in “Clinical utility of plasma amyloid beta biomarker: a multicenter validation study (CUPAB)” (jRCTs032200043); in this trial, the performance of a plasma amyloid biomarker [9] was evaluated using visual interpretation of flutemetamol PET images as the standard. In addition, this study aimed to examine the correlation between the CL values of cases with discrepancies among the experts and the reading confidence of each assessor.

## Materials and methods

### Participants

The participants were recruited from three facilities: the National Center for Geriatrics and Gerontology (NCGG), Aichi (*n* = 107); Kindai University (*n* = 65); and Tokyo Metropolitan Institute for Geriatrics and Gerontology (TMIG) (*n* = 20). The participants were then clinically categorized into the following groups: cognitively normal (CN) (*n* = 59), mild cognitive impairment (MCI) (*n* = 65), AD (*n* = 55), and non-AD dementia (non-AD) (*n* = 13).

The participants were enrolled in the CUPAB project (Public Database Registration number: jRCTs032200043, initial CRB approval date: March 25, 2020), which was approved by the Ethics Committees of all three facilities, and all participants provided informed consent.

The study adhered to the principles outlined in the Declaration of Helsinki and followed the “Ethical Guidelines for Medical and Health Research Involving Human Subjects” issued by the Ministry of Health, Labour, and Welfare in Japan.

All participants completed neuropsychological testing according to the protocol, which was described in detail previously [10].

### PET images

Aβ PET imaging with 18F-flutemetamol and magnetic resonance imaging (MRI) scans were performed at four facilities, the NCGG, Nagoya PET Imaging Center, Kindai University, and TMIG, in accordance with the study protocol for all participants, as described in detail previously [10].

Using the same imaging protocol, images were obtained over a 20-min period starting 90 min after intravenous injection, with four frames acquired every 5 min. Motion correction was subsequently conducted, the four frames were averaged, and the resulting averaged image data were used to calculate the CL value.

### Centiloid scale

PET imaging data from three facilities were sent to the NCGG and processed using the CapAIBL pipeline, innovative software developed by the Australian eHealth Research Centre, CSIRO (https://milxcloud.csiro.au/), which serves as an advanced tool for simplifying and standardizing the quantification of imaging biomarkers (CL value) without the need for MRI scans, as described previously in detail [10] [11] [12] [13] [14]. The CL calculations were performed on the computer system in NCGG running the CapAIBL processing pipeline. The regions of interest included in the calculation were the frontal, temporal, and parietal lobes and the precuneus. The reference region was the whole cerebellum [12]. The quality of the process, including spatial normalization, was monitored remotely by CSIRO.

### Raters

The expert raters (*n* = 3) were nuclear medicine physicians licensed in Japan who had undergone tracer-specific reading training supplied by GE Healthcare. All the expert raters had between 2 and 4 years of experience in reading flutemetamol PET images.

### Expert visual rating procedure

Online visual rating platforms, IRUM ^®^ neo Report software (Micron Inc., Tokyo, Japan) and EV Insite ^®^ R Viewer software (PSP Corporation, Tokyo, Japan) have been developed to enable expert raters at multiple facilities to access the platform remotely. The visual ratings were performed over seven sessions, which were conducted over a period of approximately 2 years.

PET images were calibrated for pons luminance to 90% of the maximum luminance using a rainbow color scale. Aβ accumulation was visually evaluated in five main regions (frontal lobe, temporal lobe, parietal lobe, posterior cingulate gyrus, and precuneus, and striatum). Patients with positive accumulation in any of the five regions were classified as Aβ-positive, and those with negative accumulation in all five regions were classified as Aβ-negative.

The confidence level of the visual assessment of amyloid accumulation by each rater was indicated on a three-point scale (neither, probable, or certain). The confidence levels were discretized as follows: neither: −1, probable: 0, and certain: + 1. The sum of these discretized confidence levels by the three raters for each case was considered the overall confidence level of the three raters, ranging from −3 to + 3.

Amyloid PET-positive or -negative classifications were made on the basis of the results of the expert raters. If there were discrepancies in expert visual ratings, the majority vote was employed as the decision. The visual ratings of the expert raters were classified into the following four groups: all negative (nnn); 1 positive, 2 negative (nnp); 2 positive, 1 negative (npp); and all positive (ppp).

## Statistical analysis

We calculated point estimates and 95% confidence intervals for interrater agreement of the visual assessments among the three experts. This analysis was performed both overall and separately for each brain region (frontal lobe, temporal lobe, parietal lobe, posterior cingulate gyrus and precuneus, and striatum) using statistical methods such as Fleiss’s kappa, Conger’s kappa, and Krippendorff’s α.

We then analyzed the CL values across the four visual rating groups using one-way analysis of variance followed by Bonferroni post hoc analysis.

We used receiver operating characteristic (ROC) curve analysis to assess the discrimination ability of the CL scale using visual judgment as the standard of reference. We evaluated the performance of the CL scale in classifying cases as completely negative (nnn vs. the three other groups (nnp, npp, and ppp)) and completely positive (ppp vs. the three other groups (npp, nnp, nnn)). In addition, we examined the discrimination ability of the CL scale between a negative majority decision (nnn, nnp) and a positive majority decision (npp, ppp). The area under the curve (AUC) was calculated to determine the optimal sensitivity and specificity using cutoff values determined by the Youden index.

We used the statistical analysis environment R version 4.2.2 and Stata 18 software (Stata 18.0; Stata Corp version, USA) for the statistical analysis.

## Results

### Agreement of expert visual ratings

The visual rating results, which were based on the majority vote of three expert raters, included 101 positive and 91 negative decisions. Of the positive decisions, there were 92 complete agreements and 9 disagreements (8.91% discrepancies); of the negative decisions, there were 75 complete agreements and 16 disagreements (17.58% discrepancies). The overall discrepancy rate was 13.02% (25/192), with a higher rate for negative decisions than for positive decisions (Table 1).

**Table 1:** Visual ratings of 3 experts

Rating (positive)	Rating (negative)	*n*	Decision	*n* (%)
0	3	75	Negative	91 (47.4%)
1	2	16		
2	1	9	Positive	101 (52.6%)
3	0	92		

The positive- or negative-amyloid PET decision was based on the results of the expert raters. When there were differences in the expert visual ratings, the majority vote was adopted as the decision

### Interrater reliability

Interrater reliability was high among the 3 expert raters (Fleiss’ kappa 0.83 (0.76–0.89), Conger’s kappa 0.83 (0.76–0.89), and Krippendorff’s α 0.83 (0.76–0.89)) (Table 2). When each brain region was examined separately, we found that the frontal lobe, posterior cingulate gyrus/precuneus, and striatum exhibited greater interrater agreement than the overall results did. In contrast, the temporal and parietal cortices demonstrated lower agreement than the overall results did. In addition, no differences were observed between the left and right sides.

**Table 2:** Interrater reliability with the Fleiss’ kappa, Conger’s kappa, and Krippendorff’s α tests

	Fleiss’ kappa	95% CI	Conger’s kappa	95% CI	Krippendorff’s α	95% CI
**Overall**	**0.83**	**(0.76 0.89)**	**0.83**	**(0.76 0.89)**	**0.83**	**(0.76 0.89)**
Frontal_lt	0.86	(0.80 0.92)	0.86	(0.80 0.92)	0.86	(0.80 0.92)
Frontal_rt	0.87	(0.81 0.93)	0.87	(0.81 0.93)	0.87	(0.81 0.93)
Temporal_lt	0.81	(0.74 0.87)	0.81	(0.74 0.87)	0.81	(0.74 0.88)
Temporal_rt	0.81	(0.74 0.88)	0.81	(0.75 0.88)	0.81	(0.74 0.88)
Parietal_lt	0.78	(0.71 0.85)	0.78	(0.72 0.85)	0.78	(0.71 0.85)
Parietal_rt	0.78	(0.71 0.85)	0.79	(0.72 0.85)	0.78	(0.72 0.86)
Posterior cingulate gyrus/precuneus _lt	0.86	(0.80 0.92)	0.86	(0.80 0.92)	0.86	(0.80 0.92)
Posterior cingulate gyrus/precuneus _rt	0.87	(0.82 0.93)	0.87	(0.82 0.93)	0.87	(0.82 0.93)
Striatum _lt	0.86	(0.80 0.92)	0.86	(0.80 0.92)	0.86	(0.80 0.92)
Striatum _rt	0.87	(0.81 0.92)	0.87	(0.81 0.92)	0.87	(0.81 0.93)

Kappa (95% CI) was calculated as kappa ± Z (1 – α/2) * SE (kappa)

Krippendorff’s α (95% CI) is calculated as Krippendorff’s α ± Z (1 – α/2) * SE (Krippendorff’s α)

*CI*: confidence interval, *se*: standard error

Representative overall results are shown in bold

### Effects of expert visual ratings obtained in multiple sessions

The expert visual ratings were obtained in seven separate sessions over a period of approximately 2 years. For each of the seven sessions, we assessed trends in the degree of disagreement among the expert raters and found that the level of disagreement remained consistent throughout the seven sessions.

### Relationships between CL values and expert visual ratings

**Fig. 1:**
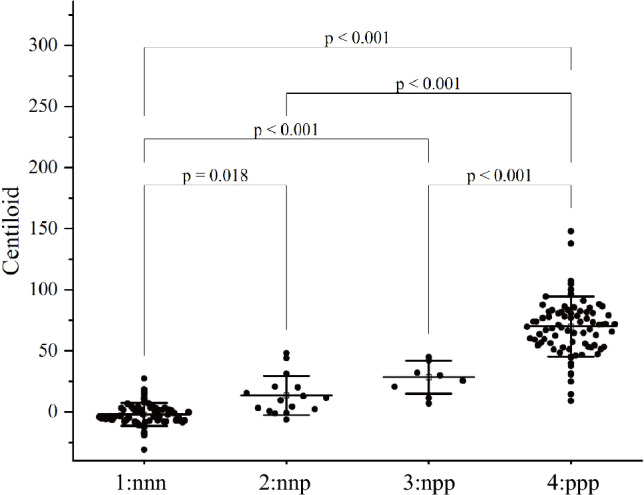
Scatter plot of the Centiloid values across the four groups. Centiloid values were plotted for each group. **a** All rated negative (nnn). **b** One rated positive and two rated negative (nnp). **c** Two rated positive and one rated negative (npp). **d** All rated positive (ppp). One-way analysis of variance followed by the Bonferroni post hoc correction was used

Table 3 and Fig. 1 illustrate the relationships between CL values and expert visual ratings across the four groups: (1) all-negative ratings (nnn); (2) one positive rating and two negative ratings (nnp); (3) two positive ratings and one negative rating (npp); and (4) all-positive ratings (ppp). The all-positive group demonstrated a significantly greater CL value than the other groups did, and the CL values of the one- and two-positive groups were significantly greater than that of the all-negative group. However, there was no significant difference between the one- and two-positive groups.

**Table 3:** Relationship between Centiloid values and expert visual ratings

	All negative	One positive	Two positive	All positive	*p* value
n	75	16	9	92	
Centiloid value (mean ± SD)	−2.0 ± 9.4	13.6 ± 16.1	28.5 ± 13.6	70.0 ± 24.6	< 0.0001†

The data are the means (standard deviation)

In the case of one positive or two positive ratings, the majority vote was defined as agreement between two of three raters

^†^ One-way analysis of variance followed by Bonferroni post hoc correction

### ROC analysis

**Fig. 2:**
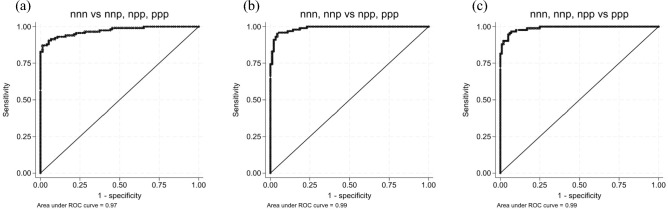
ROC analyses of interrater variability. Four patterns of majority decision-making by the three raters were observed: unanimous positive (ppp), majority positive (npp), majority negative (nnp), and unanimous negative (nnn). (**a**) ROC analysis of the discriminative ability of the CL value to distinguish between the unanimous negative (nnn) group and the other groups (nnp, npp, and ppp). ROC analysis revealed an AUC of 0.97, a cutoff value of 18.26, and a sensitivity/specificity of 0.87/0.99. (**b**) ROC analysis of the discriminative ability of the CL value to distinguish between the nnn and nnp groups vs. the npp and ppp groups. ROC analysis revealed an AUC of 0.99, a cutoff value of 20.8, and a sensitivity/specificity of 0.96/0.95. (**c**) ROC analysis of the discriminative ability of the CL value to distinguish between the unanimous positive (ppp) group and the other groups (nnn, nnp, and npp). ROC analysis revealed an AUC of 0.99, a cutoff value of 29.87, and a sensitivity/specificity of 0.97/0.95

We performed an ROC analysis to distinguish between the all-negative (nnn) group and the three other groups (nnp, npp, and ppp), as shown in Fig. 2a. The resulting AUC was 0.97, with a cutoff value of 18.26. The sensitivity and specificity were 0.87 and 0.99, respectively. Another ROC analysis was performed to examine the discrimination ability in cases visually classified as negative (nnn, nnp) vs. cases classified as positive (npp, ppp), as shown in Fig. 2b. The AUC was 0.99, with a cutoff value of 20.8. The sensitivity and specificity were 0.96 and 0.95, respectively. Finally, an ROC analysis was performed to compare the all-positive group (ppp) with the three other groups (nnn, nnp, and npp), as shown in Fig. 2c. The AUC was 0.99, with a cutoff value of 29.87. The sensitivity and specificity were 0.97 and 0.93, respectively.

### Relationship between the CL value and confidence in expert visual ratings

**Fig. 3:**
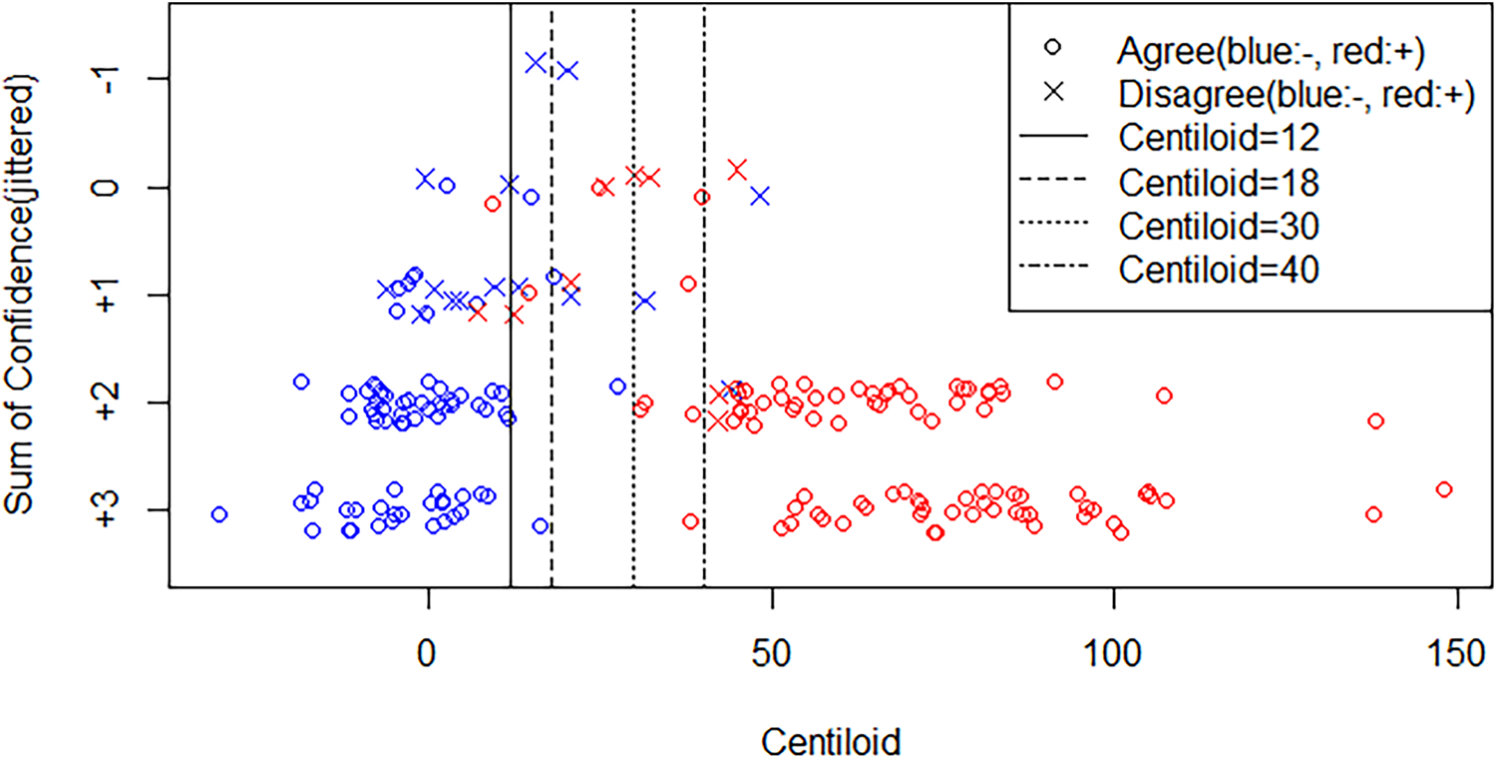
Relationships between the sum of confidence scores, Centiloid values, and expert visual ratings. The confidence levels were discretized as follows: neither: −1, probable: 0, and certain: + 1. The sum of the scores assigned by the three interpreters was considered the confidence level for each case. For example, if all three raters reported a confidence level of + 1 in a case, the total confidence level was + 3 for that case. The vertical axis is the sum of confidence levels, the horizontal axis is the Centiloid value, and the relationship between confidence and the Centiloid scale is plotted. The circles indicate agreement cases, the crosses indicate disagreement cases, red indicates amyloid PET-positive cases, and blue indicates amyloid PET-negative cases

The raters assigned confidence scores (−1 for neither, 0 for probable, + 1 for certain) to each case. The relationships among the sum of the confidence scores, CL values, and expert visual ratings are depicted in Fig. 3 and Table 4. In cases with high confidence (score + 3), the three raters exhibited perfect agreement for both negative and positive readings. However, as the total confidence level decreased, the number of discrepancies (one positive reading and two negative readings or one negative reading and two positive readings) increased among the three raters.

**Table 4:** Distribution of cases categorized by the Centiloid value

	Sum of confidence					Total, *n*
Categorized by Centiloid value	+ 3	+ 2	+ 1	0	−1	
Centiloid value < 12	26 (0)	38 (3)	14 (50)	4 (50)	0	82 (12)
12 < = Centiloid value < 18	1 (0)	0	3 (67)	1 (0)	1 (100)	6 (50)
18 < = Centiloid value < 30	0	1 (0)	3 (67)	3 (67)	1 (100)	8 (62)
30 < = Centiloid value < 40	1 (0)	3 (0)	2 (50)	2 (50)	0	8 (25)
40 < = Centiloid value	43 (0)	43 (7)	0	2 (100)	0	88 (6)
Total, n	71 (0)	85 (5)	22 (55)	12 (58)	2 (100)	192 (13)

Parentheses show percentage of cases with discrepancies in the judgment between the three raters

The raters assigned confidence scores (−1 for neither, 0 for probable, + 1 for certain) to each case. In this study, there were no cases in which the sum of confidence scores was −2 or −3

The cutoff values obtained with the ROC analyses in this study for all-negative vs. the three other groups and all-positive vs. the three other groups were approximately 18 and 30 CL, respectively. The gray zone for this study was predicted to be between 18 and 30 CL, with no cases at a high confidence level (score + 3) falling within that range. At the highest confidence level (score + 3), there are no cases of discrepancy among the raters. Conversely, as the confidence level decreased, the number of cases with CL values between 18 and 30 tended to increase. Furthermore, as the level of confidence decreased, the number of cases where the ratings were not in agreement increased: in more than half of the cases where the sum of confidence levels was lower than + 1, the ratings did not match.

## Discussion

In the present study, the interrater agreement among the three expert raters, measured by Fleiss’ kappa, was 0.83 (0.76–0.89), indicating a particularly high level of agreement [15]. The lower limit of the 95% confidence interval was above 0.61, and the upper limit of the 95% confidence interval was above 0.81, suggesting that, as an interval estimate, interrater reliability was substantial to almost perfect. The discrepancy rate was 13%, with 25 discrepancies out of 192 cases.

Numerous studies have investigated the correlation between visual interpretation of amyloid PET scans and quantitative analysis [16] [17] [18] [19] [20] [21]. The assessment discrepancy rate in our study was comparable to those of previous studies, ranging from approximately 9% to 38%. In these cases, the CL value for quantitative evaluation exhibited intermediate values between fully negative and fully positive.

In a previous international multicenter study conducted by Bischof et al. [4], four experts and three non-experts interpreted amyloid images acquired using three types of tracers, 18F-florbetaben, 18F-florbetapir and 18F-flutemetamol, and assigned a binary rating of positive or negative. A high degree of agreement was observed among the experts, but a significant discrepancy was observed among the non-experts.

The Krippendorff’s α values of interrater agreement were 0.68, 0.79, and 0.75 for 18F-florbetaben, 18F-florbetapir, and 18F-flutemetamol, respectively. These values indicated that the visual interpretation of amyloid imaging could be well standardized and did not depend on the visual rating protocol for each tracer for the expert readers.

Similar to previous studies, the degree of agreement in this study was calculated using Krippendorff’s α, and a value of 0.83 was obtained. Despite differences in the number of cases and raters and the ratio of positive to negative cases, the agreement in this study was the same as, or even greater than, the agreement reported previously by four experts. The study by Bischof et al. also incorporated a confidence level for each assessment, and the observed trend of high confidence for matched cases and low confidence for discrepant cases was consistent with the findings of the current study.

Various reports on the cutoff values of CL for amyloid PET have been published. Recently, Pemberton et al. reported the following cutoff values for CL related to neuropathology: a CL of less than 10 indicates that neuritic plaques are not present and that AD is negative. Conversely, a score above 50 strongly suggests AD. A score of 20 or higher indicates at least moderate plaque density [22] [23]. Salvadó G et al. [24] compared Aβ42 levels in cerebrospinal fluid with amyloid PET imaging and identified a CL cutoff of 12 or less, which is consistent with existing reports derived from postmortem neuropathological indices in AD patients.

In the AHEAD3-45 clinical trial, a CL value of 20–40 was categorized as pathologically moderate, whereas a CL value of 40 or higher was considered indicative of pathological conditions; specifically, the level of amyloid was associated with a risk of tau spreading beyond the medial temporal cortex on tau PET and coincided with the visual amyloid read cutoff for amyloid positivity [25].

In this study, the number of cases with a CL value between 18 and 30 increased as the confidence level decreased, and cases with CL values of 18–30 and higher confidence levels were not common. Table 4 shows the percentage of disagreement, which is the percentage of cases with discrepancies in the visual readings of amyloid PET images between the three raters and tended to increase at a lower sum of confidence levels. Although the discrepancy percentage is expected to increase when confidence is lower, notably high discordance rates were also observed within the borderline range of CL 18–30, which is within the borderline zone of CL 12–40 that has been presented in previous studies [24] [25]. In addition, the cutoff value for overall negative/positive discrimination, including majority judgment, was 20.8, which is consistent with the cutoff value reported by Pemberton et al., indicating at least moderate plaque presence [22]. CL 20–40 is pathologically classified as moderate, meaning that amyloid accumulation is mild; therefore, naturally, only a small amount of amyloid accumulation is detected by PET, and visual evaluation is difficult.

Figure 3 shows that among the cases with low confidence (score + 1 or 0) outside the gray zone range, more cases were negative than positive, with no agreement in the evaluation. Amyloid PET assesses the frontal lobe, posterior cingulate gyrus/precuneus, lateral temporal lobe, parietal lobe, and striatum. If any of these areas are positive, the overall result is considered positive. In cases with a high degree of certainty, most of these sites are likely positive, making the diagnosis straightforward. Conversely, in negative cases, it is necessary to confirm that none of these areas are positive, which takes more time and is more complex than a positive determination, where identifying just one positive area is sufficient. This difference in diagnostic methods is considered one of the factors contributing to discrepant results.

To improve inter-reader agreement, it is important to initially review the signs and artifacts associated with local accumulation of the amyloid PET tracer [26]. In this study, the inter-reader agreement rate for the parietal and temporal lobes was relatively low. Establishing points of focus for negative and positive visual findings in these regions may lead to improvement in the agreement rate. A combination of visual reading, quantitative assessment using the CL scale, and the use of automated analysis tools [21] [27] may be beneficial for accurate diagnosis. Artificial intelligence may have the potential to improve this process. Several studies have reported the use of deep learning (DL) models to determine amyloid positivity/negativity.

Recently, Fan et al. presented a DL model that achieved high diagnostic accuracy with good generalization across various datasets and tracers [28]. Son et al. reported on 54 cases that were judged equivocal by visual assessment. Among these 54 suspicious cases, the agreement between the DL model and visual assessment was low, with negative cases (agreement rate of 13%) showing a greater discrepancy than positive cases (agreement rate of 56%) [29].

Thus, discrepancies between DL and visual assessments are likely to occur in cases where visual assessment is equivocal. One of the benefits of DL is its potential to provide consistency and reduce variability in radiologists’ interpretations. However, the performance of DL models depends on the quality of the training data. If the model is trained on low-quality data, there is a risk of misdiagnosis. Therefore, we believe that it is necessary to create high-quality training data, especially for equivocal cases.

This study has several limitations. First, this study focused solely on the evaluation of 18F-flutemetamol images and did not assess other amyloid PET tracers. Second, owing to the research nature of the study, the subjects included individuals with normal cognitive function and those with severely impaired cognitive function. This breakdown of subjects may differ from the spectrum encountered in actual clinical practice. Amyloid PET data in real-world clinical settings are anticipated to involve a greater frequency of patients with MCI than that in this study, which included cognitively unimpaired patients, potentially resulting in a greater occurrence of equivocal cases.

Finally, it should be noted that the CL scale itself has limitations. One of the drawbacks of the CL scale is that the occipital lobe is not included in the regions of interest for CL calculation. In other words, if amyloid accumulates mainly in the occipital lobe, this accumulation is not reflected in the CL value. A pattern of amyloid deposition that first accumulates in the occipital lobe and then spreads from that lobe has been reported previously [30].

In addition, the CL value contains a certain kind of error. CL is calculated by linearly transforming the standard uptake value ratio (SUVR). Therefore, the error of the SUVR is directly included in the error of the CL. The SUVR and non-displaceable binding potential (BPND) [31] have been compared as quantitative measures of amyloid PET accumulation. BPND is obtained by kinetic analysis of dynamic data continuously collected immediately after the administration of an amyloid PET tracer (e.g., 0–110 min after administration). In contrast, the SUVR is calculated from static images collected 90–110 min after administration. The SUVR images are equivalent to the static images used for visual reading. The SUVR overestimates the amyloid burden compared with BPND [31]. In addition, the inter-reader agreement rate in the visual readings of each image is lower for SUVR images than for BPND images [32]. These findings suggest that the values or images based on the SUVR, namely, the CL, are not ideal surrogates of amyloid PET tracer accumulation.

## Conclusion

In summary, three experts independently evaluated amyloid PET images of 192 patients and demonstrated a high level of interrater agreement. Cases with low confidence levels had poorer agreement and intermediate CL values. In patients with intermediate levels of amyloid accumulation on PET imaging, caution should be exercised when making judgments.

## Data Availability

The dataset can be requested from the principal investigator of the CUPAB study (A. N.) upon reasonable request.
